# Large Duodenal Hematoma Causing an Ileus after an Endoscopic Duodenal Biopsy in a 6-Year-Old Child: A Case Report

**DOI:** 10.3390/medicina58010012

**Published:** 2021-12-22

**Authors:** Benjamin Schiller, Michael Radke, Christina Hauenstein, Carsten Müller, Christian Spang, Daniel A. Reuter, Jan Däbritz, Johannes Ehler

**Affiliations:** 1 Department of Pediatrics, Rostock University Medical Center, 18057 Rostock, Germany; benjamin.schiller@med.uni-rostock.de (B.S.); michael.radke@med.uni-rostock.de (M.R.); jan.daebritz@med.uni-rostock.de (J.D.); 2 Institute of Diagnostic and Interventional Radiology, Pediatric Radiology and Interventional Radiology, Rostock University Medical Center, 18057 Rostock, Germany; christina.hauenstein@med.uni-rostock.de; 3 Department of Pediatric Surgery, Rostock University Medical Center, 18057 Rostock, Germany; carsten.mueller@med.uni-rostock.de; 4 Department of Anesthesiology and Intensive Care Medicine, Interdisciplinary Pediatric Intensive Care Medicine, Rostock University Medical Center, 18057 Rostock, Germany; christian.spang@med.uni-rostock.de (C.S.); daniel.reuter@med.uni-rostock.de (D.A.R.)

**Keywords:** intramural duodenal hematoma, ileus, endoscopy, pediatrics, complications, case report

## Abstract

Intramural duodenal hematoma (IDH) in children is a rare complication after esophagogastroduodenoscopy. It is commonly described in patients with additional disorders or risk factors, such as coagulopathy. We present a case of a previously healthy 6-year-old boy with a large obstructing intramural duodenal hematoma and concomitant pancreatitis after an elective esophagogastroduodenoscopy. The patient presented with typical symptoms of an IDH, such as abdominal pain and distension, nausea and vomiting. IDH was diagnosed using ultrasound and magnetic resonance imaging examination. Conservative management with gastric decompression using a nasogastric feeding tube, bowel rest, total parenteral nutrition and analgesia was performed. After three weeks, the patient was discharged from the hospital without any complaints. Interventional management of IDH in pediatric patients with a lack of response to conservative therapy or complicating IDH should be discussed in an interdisciplinary team.

## 1. Introduction

An intramural duodenal hematoma (IDH) is a very rare diagnosis in healthy children and adolescents. The literature describes divergent risk factors and causes for the formation of an IDH, with children being more often affected than adults. In children and adolescents, blunt abdominal trauma, child abuse and endoscopies in patients with severe disorders are common causes [1]. Likewise, more rare causes, such as a spontaneous intramural duodenal hematoma as the manifestation of Noonan syndrome are possible [2]. In adults, trauma [3] and biopsies [4] are also described as causes of an IDH, but various other reasons and risk factors are mentioned, such as percutaneous endoscopic gastrostomy [5], endoscopic injection therapy of a bleeding ulcer [6,7], an anticoagulating therapy [8], coagulopathy (e.g., Willebrand disease) [9] or an IDH as a complication of alcohol-induced pancreatitis [10,11]. In this article, we report a rare case of a large intramural duodenal hematoma following elective endoscopic duodenal biopsy to exclude celiac disease in a previously healthy 6-year-old boy.

## 2. Methods

The case report was drawn up according to the CARE Guidelines [12]. The CARE Checklist is attached as a Supplementary File S1 [13].

## 3. Case Report

A 6-year-old boy presented with abdominal pain, abdominal distension, nausea and persistent vomiting two days after an elective endoscopic duodenal biopsy.

The endoscopy was performed in analgosedation to exclude a celiac disease since the patient occasionally complained about dizziness, nausea and abdominal pain in combination with elevated deamidated gliadin peptides and the evidence of the heterodimeric surface receptors HLA-DQ2 and -DQ8, although IgA class antibodies against transglutaminase 2 were normal. The endoscopy was performed by a very experienced pediatric gastroenterologist without periprocedural abnormalities. Three duodenal biopsies were obtained using a super-slim videogastroscope Olympus GIF-XP 190N (outer diameter 5.4 mm) with the Olympus EndoJaw FB-241K oval fenestrated biopsy forceps with a maximum insertion portion diameter of 1.9 mm. The endoscopic assessment revealed a visually normal mucosa of the upper gastrointestinal tract without any sign of augmented bleeding due to the biopsies. Histological examination confirmed the completely inconspicuous duodenal mucosa, thus celiac disease was excluded. In the patient’s medical history, neither signs of bleeding disorders nor any anticoagulant drugs were present.

The physical examination revealed a reduced general condition with fatigue, pain-related immobilization, cold sweats, pale skin color, tachycardia, high-pitched bowel sounds, abdominal distension and abdominal tenderness with punctum maximum in the upper abdomen.

**Figure 1:**
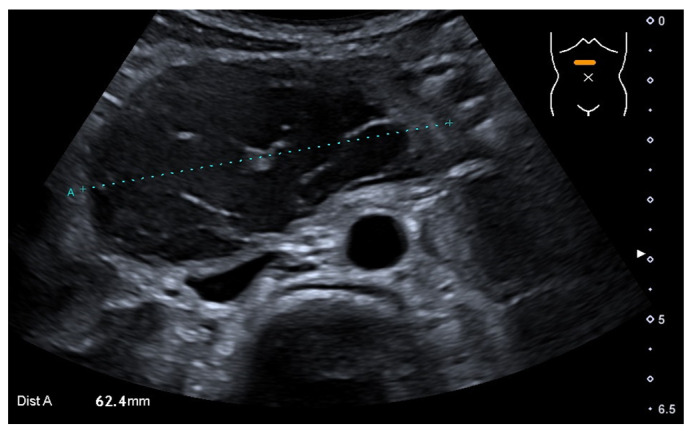
Initial abdominal ultrasound with intramural hematoma of the duodenum (dotted line), which completely compressed its lumen.

**Figure 2:**
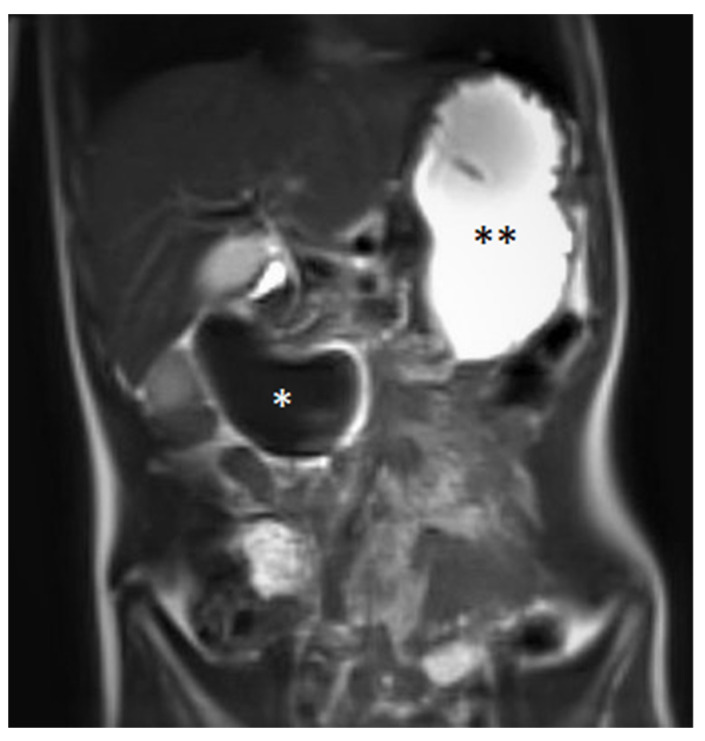
Coronal magnetic resonance imaging in the T2 HASTE sequence showing a large duodenal hematoma (*) and an ileus of the proximal small intestine with the filled stomach (**). Image quality is reduced due to the patient being restless.

A blood examination, including hemoglobin value (9.2 mmol/L—normal value (nv): 7.4–9.1 mmol/L), platelet count (441 Gpt/L—nv: 150–530 Gpt/L), prothrombin time (94%—nv: 70–130%), partial thromboplastin time (24.1 s—nv: 27–40 s) and the international normalized ratio (1.03—nv: 0.8–1.25) gave no evidence of impaired blood clotting or anemia. Prior to the procedure, no further coagulation diagnostics were available due to the inconspicuous medical and family history of bleeding. The initial ultrasound (Figure 1) examination revealed the suspicion of a large IDH causing a mechanic ileus of the proximal small intestine. The subsequently performed magnetic resonance imaging (MRI, Figure 2) confirmed the suspected diagnosis—a large IDH in the area of the duodenal C with an extent of 62 × 36 × 35 mm that completely compressed the intestinal lumen in the sense of mechanical ileus.

Based on an interdisciplinary discussion between pediatric gastroenterology, pediatric surgery, pediatric radiology and pediatric intensive care, we chose non-surgical management with gastric decompression via a nasogastric feeding tube, bowel rest, total parenteral nutrition via central venous catheter and analgesics. Fortunately, the patient’s general condition consistently improved using this therapeutic approach. Regular ultrasound examinations gave evidence for continuous septation, organization and resorption of the hematoma and resolution of the intestinal obstruction. Regularly arranged blood tests confirmed normal values for platelet count and blood clotting. Furthermore, the patient never developed anemia that would have required transfusion. After seven days, a subtle transport of fluid into the distal intestine could be detected. We were able to restart the enteral nutrition 18 days after hospital admission, with sonographic and clinical evidence of a continuous intestinal transport of even larger amounts of fluid. On day 19, the hematoma was no longer visible using ultrasound examination. The patient was discharged from the hospital 26 days after admission without any complaints and with full enteral nutrition.

As a secondary finding, the IDH initially led to slight consecutive congestion of the common and hepatic bile ducts. This subtle cholestasis with accompanying pancreatitis was confirmed via ultrasound and laboratory tests (maximum values of pancreas lipase (351 U/L—nv: <57 U/L), alpha-amylase (117 U/L—nv: 28–100 U/L), aspartate-aminotransferase (115 U/L—nv: <45 U/L), alanine-aminotransferase (110 U/L—nv: <40 U/L) and gamma-glutamyltransferase (30.6 U/L—nv: 27 U/L). All mentioned laboratory parameters continuously decreased and normalized until day 18, in line with the resorption of the IDH.

## 4. Discussion

An IDH in healthy children is a very rare post-endoscopic complication, in contrast to patients with additional disorders, who are more susceptible to bleedings, perforations and mucosal tears [14]. IDH in children is commonly caused by blunt abdominal trauma or child abuse [15]. To date, the post-endoscopic incidence of IDH cannot be derived from well-powered observational studies, as predominantly case reports and thematic reviews exist. Available literature reports vague incidences between zero in 9308 examinations [14] up one in 1922 procedures [16]. In children with severe disorders, e.g., after organ or bone marrow transplant, the risk of complications, such as an IDH, is further increased [16,17,18].

Symptoms of an IDH, which typically present within three days after biopsy, are abdominal pain, abdominal tenderness, vomiting and anemia [16,17]. As presented in our case, the most common complications of post-endoscopic IDH are bowel obstruction with the need for total parental nutrition (43% of IDH cases), as well as pancreatitis (43 to 56% of IDH cases) and biliary tract compression in approximately 29% of IDH cases [16,17]. However, due to the rarity of a post-endoscopic IDH and the missing prospective data, the incidence is of limited significance. With clinical suspicion of a duodenal hematoma, ultrasound or MRI should be performed to verify the diagnosis and to exclude differential diagnoses, such as a duodenal duplication, abscess, pancreatic pseudocyst or solid tumor [19]. A computed tomography (CT) is also described in several case reports, but MRI is superior due to the lack of ionizing radiation exposure [20] and superior soft-tissue resolution. As in our case, an IDH is mostly located in the first to third parts of the duodenum [21]. The pathogenesis is not clear, though some authors discuss the fixation of the duodenum and the abundant vascularization of the submucosa as a possible explanation [22,23]. Others refer to the shear-off of the mucosa from the submucosa by the biopsy as a conceivable cause [21,24]. However, this contradicts the assumption that the risk for developing an IDH seems to be independent of the number of biopsies taken during an endoscopy [16].

In the histopathologic assessment of the biopsy material, no transected or lacerated blood vessels were found. The overall tissue lesion could be minimized by using particularly small biopsy forceps. Furthermore, there was no sign of augmented periprocedural bleeding. Along with this, the further clinical and coagulation monitoring gave no evidence for an increased bleeding tendency. Hence, neither a direct vascular lesion nor an undetected bleeding disorder seemed to be the likely cause of the IDH in the presented case. We suspect the shear-off of the mucosa from the submucosa to be the most suitable reason.

Due to the rarity of an IDH in pediatric patients, therapeutic management should be discussed in an interdisciplinary team.

Conservative management with bowel rest, total parenteral nutrition and analgesics results in a good outcome with low mortality, as described in the available literature [3,17,19,25,26,27]. An interventional therapeutic approach may give rise to risks of additional complications and should be reserved for cases with treatment failure of conservative management; complicated obstructing IDH, e.g., with severe pancreatitis; signs of acute intra-abdominal hemorrhage; infarction; and peritonitis [28,29]. Depending on the characteristics and location of the IDH, various procedures are available. These comprise endoscopic incision and drainage [28], percutaneous drainage [29,30], laparoscopic drainage [31] and endoscopic balloon catheter dilatation [32,33].

Repeated clinical and ultrasound examinations are recommended for follow-ups, which should include the monitoring of IDH size and characteristics, as well as the degree of duodenal compression [19,20,34]. In the majority of patients, the time to IDH resolution ranges from one to three weeks, which is in line with the case presented here [27,34]. The overall mortality of IDH is reported to be low, with a margin from 0 to 1 in 2000 cases [35].

The main reasons for the preferred conservative management were a patient in a stable condition and the possibility of close clinical (intensive care), sonographic and laboratory monitoring. Based on the available literature, we assumed a good outcome from conservative management in children with post-endoscopic IDH. Given the rarity of pediatric IDH following endoscopy, there is still a lack of evidence for a better outcome achieved by surgical or interventional management. Furthermore, several risks of interventional approaches (e.g., general risk of anesthesia, infections, bleedings, necrosis, injury of enclosed organs, fistulas, hernias and wound healing disorders) favored a conservative strategy due to risk–benefit analysis.

In the case of deterioration of the patients’ clinical condition or evidence of a significant increase in the size of the IDH, we would have favored a minimally invasive procedure, such as ultrasound-assisted endoscopic or percutaneous drainage, and not primarily a surgical intervention. However, as there is currently no evidence for one approach with a better outcome, from the authors’ point of view, children should be treated with a minimally invasive intervention.

In conclusion, of the existing literature regarding the low overall mortality and the higher risks of interventions, conservative management of IDH should be preferred as first-line therapy.

## 5. Conclusions

New abdominal symptoms following an upper gastrointestinal tract biopsy need an immediate diagnostic workup. In addition to a detailed medical history and clinical examination, blood sampling and non-ionizing imaging, such as ultrasound and/or MRI, should be initiated. An obstructing intestinal hematoma is a very rare complication of an upper endoscopy. Regardless of the etiology, conservative management with gastric decompression, bowel rest, total parenteral nutrition and analgesics has a good outcome with low mortality. Based on interdisciplinary discussion, interventional management might be an option in severe cases.

## Data Availability

All data supporting the study are included in the manuscript.

## Supplementary Materials

The following are available online at https://www.mdpi.com/article/10.3390/medicina58010012/s1, File S1: Checklist according to the CARE-guidelines [12,13].
