# Supplementary material for: Large Duodenal Hematoma Causing an Ileus after an Endoscopic Duodenal Biopsy in a 6-Year-Old Child: A Case Report
Source: Medicina (Kaunas). 2021 Dec 22;58(1):12. doi: 10.3390/medicina58010012 (PMC8780155; doi:10.3390/medicina58010012)
Supplement: Supplementary file 1 [file medicina-58-00012-s001.zip › medicina-1475155-supplementary.pdf]

Checklist according to the CARE-guidelines [12,13]

Topic	Item	Checklist item description	Reported on page
**Title**	**1**	The diagnosis or intervention of primary focus followed by the words “case report“	1
**Key Words**	**2**	2 to 5 key words that identify diagnoses or interventions in this case report, including "case report"	1
**Abstract (no references)**	**3a**	Introduction: What is unique about this case and what does it add to the scientific literature?	1
	**3b**	Main symptoms and/or important clinical findings	1
	**3c**	The main diagnoses, therapeutic interventions, and outcomes	1
	**3d**	Conclusion—What is the main “take-away” lesson(s) from this case?	1
**Introduction**	**4**	One or two paragraphs summarizing why this case is unique (**may include** references)	1
**Patient information**	**5a**	De-identified patient specific information.	2
	**5b**	Primary concerns and symptoms of the patient	2
	**5c**	Medical, family, and psycho-social history including relevant genetic information	2
	**5d**	Relevant past interventions with outcomes	2
**Clinical findings**	**6**	Describe significant physical examination (PE) and important clinical findings	2
**Timeline**	**7**	Historical and current information from this episode of care organized as a timeline	N/A
**Diagnostic assessement**	**8a**	Diagnostic testing (such as PE, laboratory testing, imaging, surveys)	2/3
	**8b**	Diagnostic challenges (such as access to testing, financial, or cultural)	3
	**8c**	Diagnosis (including other diagnoses considered)	2/3
	**8d**	Prognosis (such as staging in oncology) where applicable	3
**Therapeutic intervention**	**9a**	Types of therapeutic intervention (such as pharmacologic, surgical, preventive, self-care)	3
	**9b**	Administration of therapeutic intervention (such as dosage, strength, duration)	3
	**9c**	Changes in therapeutic intervention (with rationale)	N/A
**Follow-up and outcomes**	**10a**	Clinician and patient-assessed outcomes (if available)	3
	**10b**	Important follow-up diagnostic and other test results	3
	**10c**	Intervention adherence and tolerability (How was this assessed?)	3
	**10d**	Adverse and unanticipated events	N/A
**Discussion**	**11a**	A scientific discussion of the strengths AND limitations associated with this case report	3-5
	**11b**	Discussion of the relevant medical literature **with references**	3-5
	**11c**	The scientific rationale for any conclusions (including assessment of possible causes)	3-5
	**11d**	The primary “take-away” lessons of this case report (without references) in a one paragraph conclusion	5
**Patient perspective**	**12**	The patient should share their perspective in one to two paragraphs on the treatment(s) they received	N/A because of patient´s age
**Informed consent**	**13**	Did the patient give informed consent? Please provide if requested	Yes
